# A novel fMRI paradigm suggests that pedaling-related brain activation is altered after stroke

**DOI:** 10.3389/fnhum.2015.00324

**Published:** 2015-06-04

**Authors:** Nutta-on Promjunyakul, Brian D. Schmit, Sheila M. Schindler-Ivens

**Affiliations:** ^1^Department of Physical Therapy, Marquette UniversityMilwaukee, WI, USA; ^2^Department of Biomedical Engineering, Marquette UniversityMilwaukee, WI, USA; ^3^Department of Physical Medicine and Rehabilitation, Medical College of WisconsinMilwaukee, WI, USA; ^4^Clinical and Translational Science Institute of Southeastern Wisconsin, Medical College of WisconsinMilwaukee, WI, USA

**Keywords:** rehabilitation, hemiparesis, hemiplegia, locomotion, lower extremity, fMRI, imaging, plasticity

## Abstract

The purpose of this study was to examine the feasibility of using functional magnetic resonance imaging (fMRI) to measure pedaling-related brain activation in individuals with stroke and age-matched controls. We also sought to identify stroke-related changes in brain activation associated with pedaling. Fourteen stroke and 12 control subjects were asked to pedal a custom, MRI-compatible device during fMRI. Subjects also performed lower limb tapping to localize brain regions involved in lower limb movement. All stroke and control subjects were able to pedal while positioned for fMRI. Two control subjects were withdrawn due to claustrophobia, and one control data set was excluded from analysis due to an incidental finding. In the stroke group, one subject was unable to enter the gantry due to excess adiposity, and one stroke data set was excluded from analysis due to excessive head motion. Consequently, 81% of subjects (12/14 stroke, 9/12 control) completed all procedures and provided valid pedaling-related fMRI data. In these subjects, head motion was ≤3 mm. In both groups, brain activation localized to the medial aspect of M1, S1, and Brodmann’s area 6 (BA6) and to the cerebellum (vermis, lobules IV, V, VIII). The location of brain activation was consistent with leg areas. Pedaling-related brain activation was apparent on both sides of the brain, with values for laterality index (LI) of –0.06 (0.20) in the stroke cortex, 0.05 (±0.06) in the control cortex, 0.29 (0.33) in the stroke cerebellum, and 0.04 (0.15) in the control cerebellum. In the stroke group, activation in the cerebellum – but not cortex – was significantly lateralized toward the damaged side of the brain (p = 0.01). The volume of pedaling-related brain activation was smaller in stroke as compared to control subjects. Differences reached statistical significance when all active regions were examined together [p = 0.03; 27,694 (9,608) μL stroke; 37,819 (9,169) μL control]. When individual regions were examined separately, reduced brain activation volume reached statistical significance in BA6 [p = 0.04; 4,350 (2,347) μL stroke; 6,938 (3,134) μL control] and cerebellum [p = 0.001; 4,591 (1,757) μL stroke; 8,381 (2,835) μL control]. Regardless of whether activated regions were examined together or separately, there were no significant between-group differences in brain activation intensity [p = 0.17; 1.30 (0.25)% stroke; 1.16 (0.20)% control]. Reduced volume in the stroke group was not observed during lower limb tapping and could not be fully attributed to differences in head motion or movement rate. There was a tendency for pedaling-related brain activation volume to increase with increasing work performed by the paretic limb during pedaling (*p* = 0.08, *r* = 0.525). Hence, the results of this study provide two original and important contributions. First, we demonstrated that pedaling can be used with fMRI to examine brain activation associated with lower limb movement in people with stroke. Unlike previous lower limb movements examined with fMRI, pedaling involves continuous, reciprocal, multijoint movement of both limbs. In this respect, pedaling has many characteristics of functional lower limb movements, such as walking. Thus, the importance of our contribution lies in the establishment of a novel paradigm that can be used to understand how the brain adapts to stroke to produce functional lower limb movements. Second, preliminary observations suggest that brain activation volume is reduced during pedaling post-stroke. Reduced brain activation volume may be due to anatomic, physiology, and/or behavioral differences between groups, but methodological issues cannot be excluded. Importantly, brain action volume post-stroke was both task-dependent and mutable, which suggests that it could be modified through rehabilitation. Future work will explore these possibilities.

## Introduction

Functional magnetic resonance imaging (fMRI) has been used extensively to investigate cortical contributions to upper limb movement in individuals with post-stroke hemiparesis. For example, fMRI has been used to examine cortical activation during paretic wrist, hand, and finger movements; in acute and chronic stroke survivors; before, during and after rehabilitation (Cramer et al., 1997, 2000, 2001; Cao et al., 1998; Marshall et al., 2000; Pariente et al., 2001; Pineiro et al., 2001; Carey et al., 2002; Small et al., 2002; Ward et al., 2003; Dong et al., 2007). The resulting body of literature has contributed to the development of a number of models explaining how the brain adapts to injury, recovery, and rehabilitation to produce upper limb movement after stroke (Johansen-Berg et al., 2002; Calautti and Baron, 2003; Fridman et al., 2004). Such models contribute to our fundamental understanding of neural adaptation and provide a framework for scientifically grounded rehabilitation interventions.

In contrast to the upper limb, fewer studies have used fMRI to examine brain activation during paretic lower limb movement. Moreover, existing studies are limited to unilateral, single joint movements of the ankle and knee (Carey et al., 2004; Dobkin et al., 2004; Luft et al., 2004, 2005; Jang et al., 2005; You et al., 2005; Kim et al., 2006; Enzinger et al., 2008, 2009; MacIntosh et al., 2008). While these paradigms provide some insight into cortical control of the lower limb post-stroke, they lack important components of functional movements of the lower limbs, many of which are bilateral, reciprocal, and multijoint. The limited use of fMRI with lower limb movements has contributed to a lack of understanding of how the stroke-affected brain adapts – or fails to adapt – to produce such movements. Lack of knowledge in this area is problematic in light of abundant literature indicating that the cerebral cortices and other regions of the brain contribute to functional lower limb movements, including uncomplicated locomotor behaviors such as treadmill and over ground walking (Petersen et al., 2001; Nielsen, 2003; Pyndt and Nielsen, 2003). This literature provides a major advance from prior understanding that cortical involvement is limited to sophisticated locomotor behaviors such as obstacle avoidance (Drew, 1993; Drew et al., 2002, 1996). Hence, the role of the cortex and other regions of the brain should be included in any framework for understanding neural control of lower limb movement after stroke.

To address this knowledge gap, we recently developed an experimental paradigm that uses fMRI to record whole brain activation during pedaling (Mehta et al., 2009). Pedaling is a useful model because it involves continuous, reciprocal, multijoint extension and flexion of both lower limbs, and therefore, shares characteristics of functional lower limb movements. It is also possible to pedal while lying supine, thus enabling the use of fMRI. In recent publications, we described and validated our methods for recording pedaling-related brain activation with fMRI in young, healthy controls (Mehta et al., 2009). We also described the location, volume, and intensity of pedaling-related brain activation in this population (Mehta et al., 2009, 2012). The next step in narrowing the knowledge gap in stroke was to determine whether our paradigm could be used with stroke survivors. We felt that the pedaling approach was promising because our group and others have used pedaling to understand other aspects of neural control of lower limb movement post-stroke (Brown and Kautz, 1998, 1999; Kautz and Brown, 1998; Schindler-Ivens et al., 2004, 2008; Kautz et al., 2006; Fuchs et al., 2011). However, we had questions as to whether stroke survivors would be able to pedal the custom-designed device with the head and torso restrained, as is required for fMRI. We were also concerned that abnormal neck and trunk posture, which is often seen after stroke, could make it difficult to position the head for fMRI. Finally, we questioned whether head motion could be adequately minimized in the presence of stroke-related movement impairments, such as hyperreflexia, increased muscle stiffness, and non-fluid movement execution. We had similar questions regarding age-matched individuals without stroke, who would be required for control experiments.

Hence, the primary aim of this study was to examine the feasibility of recording brain activation with fMRI during pedaling in individuals with stroke and age-matched controls. Secondary aims were to identify regions of pedaling-related brain activation and to measure the volume and intensity of activation in these regions in order to identify, if present, any stroke-related changes. Portions of this work have been reported previously in abstract (Promjunyakul et al., 2011).

## Materials and Methods

### Subjects

To be included, all subjects had to be free from contraindications to fMRI (e.g., metal implants and foreign bodies, pregnancy, history of claustrophobia) and orthopedic conditions that could interfere with pedaling (e.g., severe muscle contracture, arthritis, or pain in the spine or extremities). It was also required that subjects be free from contraindications to exercise, as light exercise was inherent to the pedaling task. Contraindications to exercise included but were not limited to hypertension, angina, abnormal ECG findings, recent myocardial infarction, heart arrhythmia, heart block, or heart failure. Finally, because pedaling was done in supine, subjects were required to tolerate supine positioning for ∼2 h.

Stroke subjects had to have sustained a stroke at least 6 months prior to testing. Cortical and subcortical strokes on either side of the brain were allowed; however, strokes had to be outside the leg area of the primary sensory (S1) and primary motor (M1) cortices (i.e., the medial aspect of the pre- and post-central gyri). Exclusion of strokes in these regions was important because prior work in young, healthy adults has shown that pedaling produces brain activation in the medial aspect of M1 and S1 (Christensen et al., 2000; Mehta et al., 2009, 2012). Hence, activation in these regions would provide support for detection of pedaling-related brain activation. Control subjects were required to be free of stroke and other neurological disease or injury. Effort was made to match control and stroke groups with respect to age and sex.

Fourteen individuals with stroke [nine females; mean (SD) age 54.5 (12.3) years] and 12 controls [six females; age 53.4 (13.1) years], all of whom met inclusion criteria, gave written informed consent according to the Declaration of Helsinki and institutional guidelines at Marquette University and the Medical College of Wisconsin. Time since stroke was 11.4 (13.0) years. Eight stroke subjects had subcortical lesions involving the internal capsule, corona radiata, basal ganglia, or thalamus. Six stroke participants had lesions affecting a portion of the cerebral cortex outside the leg area of M1 and S1. There were six subjects with left- and seven subjects with right-sided stroke. One subject had subcortical lesions on the right and left side. See **Table 1**.

**Table 1:** Descriptive characteristics of stroke subjects.

ID	Age (yrs)	Sex	Side of stroke	Affected brain area	Lesion size (μL)	Mechanism of stroke	Time since stroke (yrs)	FM_LE_total/motor/sensory max = 56/44/12	Walking velocity (m/s)	%Work(+)/(-)/(net)	TSR/SLR	KINSYM (+)/(-)/(net)
S01	60	F	R	Cort	139,120	I, E	20.4	39/37/2	1.10	41.23/60.74/-3.79	0.98/1.03	47.7/66.5/-111.9
S03	62	F	L	Subcort	157	I	8.4	54/42/12	1.11	36.10/83.24/8.28	1.11/0.96	45.4/51.5/-35.8
S05	56	M	L	Subcort	51,284	H, AVM	51.0	43/31/12	1.04	35.66/90.63/24.50	1.35/1.12	31.1/66.7/-297.9
S06	64	F	R	Subcort	715	H	6.5	54/42/12	0.82	45.22/49.79/37.55	1.07/1.28	52.6/53.8/85.9
S07	20	F	L	Subcort	7,623	U	19.0	47/35/12	1.13	42.11/59.05/33.85	1.40/0.98	33.8/50.6/197.9
S08	73	F	R	Subcort	156	I, E	1.1	52/40/12	1.04	46.94/57.18/40.26	1.01/1.12	40.3/62.9/-682.2
S10	58	F	L	Cort	40,823	I, CVOD	6.1	42/30/12	0.48	40.24/68.10/-4.34	2.06/1.41	21.0/65.5/-130.9
S11	53	F	R	Subcort	600	I	17.4	53/41/12	1.05	42.95/57.80/35.65	1.16/1.07	38.6/62.8/415.5
S13	46	M	R, L	Subcort	1,518	I	4.4	37/25/12	0.82	29.87/73.81/-2.22	1.35/1.00	36.8/74.1/567.3
S14^1^	52	F	L	Cort	92,263	H, ICAD	3.0	45/33/12	0.59	NA	NA	NA
S15	48	M	R	Cort	74,433	H, ICAD	8.1	37/25/12	0.88	30.85/69.21/-40.07	2.18/1.33	7.6/57.0/-360.8
S16^2^	51	M	R	Subcort	NA	I	2.0	44/32/12	0.48	NA	NA	NA
S17	65	F	L	Cort	52,811	I	6.2	26/24/2	0.20	36.88/67.38/-4.20	1.87/4.91	NA
S19	55	M	R	Cort	136,980	I, CVOD	6.4	53/41/12	1.22	38.28/72.92/7.59	1.00/1.00	52.3/46.2/135.1
**Mean (SD)**	**54.5 (12.3)**				**46,037 (51,449)**		**11.4 (13.0)**	**44.6/34.1/10.6 (8.1)/(6.6)/(3.6)**	**0.85 (0.31)**	**38.86/67.49/11.09 (5.3)/(11.6)/(24.0)**	**1.38/1.43 (0.4)/(1.1)**	**37.0/59.8/-19.8 (13.6)/(8.5)/(356.4)**

*^1^Functional magnetic resonance imaging (fMRI) data excluded from analysis due to excessive head motion. ^2^Unable to complete fMRI due to body type incompatible with MRI. NA, Not available; F, female; M, male; R, right; L, left; Cort, stroke affecting cerebral cortex; Subcort, stroke affecting subcortical white matter; I, ischemia; E, embolism; H, hemorrhage; AVM, arteriovenous malformation; U, unknown; CVOD, cerebrovascular occlusive disease; ICAD, internal carotid artery dissection; %Work, percent work done by paretic limb during pedaling; TSR, temporal symmetry ratio during walking; SLR, spatial symmetry ratio during walking; KINSYM, symmetry of walking kinetics.*

### Procedures

Stroke subjects underwent a battery of assessments to characterize sensory and motor impairment of the lower limbs. Values are reported in **Table 1**. Tests included the 8 m comfortable walk test for walking velocity and the lower extremity Fugl-Meyer (FM_LEtotal_), which was subdivided into motor (FM_LEmotor_) and sensory (FM_LEsens_) components. FM_LEmotor_ included tests for reflex activity, synergy, coordination, and balance. FM_LEsens_ included light touch and proprioception.

We also quantified the percent mechanical work performed by the paretic limb during pedaling (%Work). This measurement was made outside the scanning environment, using a non-fMRI-compatible pedaling device equipped with force and position sensors. This device and the methods for quantifying work have been described previously (Kautz and Brown, 1998; Schindler-Ivens et al., 2008; Fuchs et al., 2011). In brief, subjects pedaled at a comfortable rate against a moderate load while we measured the forces applied to each pedal and the position of the crank and the pedals. We computed the forces oriented tangentially to the crank arm, as these forces create a torque about the crank center (referred to as crank torque) that produces angular acceleration and deceleration of the crank. Crank torque was plotted against crank angle, and the area under the resulting curve yielded the mechanical work done by the limb. Positive and negative areas were computed separately to measure the propulsive (positive area) and retarding (negative area) work done by each limb. Percent work done by the paretic limb [propulsive [%Work(+)], retardant [%Work(-)], and net [%Work(net)]] were computed as the Work(paretic)/Work(total) *100, where Work(paretic) was the work done by the paretic limb and Work(total) was the sum of the work done by both legs. Hence, 50% indicates equal sharing of the work between the paretic and non-paretic limbs, as is typical for able-bodied individuals.

Finally, we characterized stroke-related walking impairment with respect to temporal, spatial, and kinetic symmetry. Measurements were made while subjects walked at a self-selected comfortable rate along a 6 m walkway. Standard motion capture procedures were used to measure swing and stance phase time and step length. Force plates were used to record anterior–posterior ground reaction forces. From these recordings, we computed the temporal symmetry ratio (TSR), step length ratio (SLR), and the between-limb symmetry of walking kinetics (KINSYM). TSR was defined as the ratio of swing phase time to the stance phase time of the paretic to the non-paretic limb. SLR was the ratio of the paretic to non-paretic step length. Values of 1 for TSR and SLR indicated perfect kinematic symmetry between the legs. KINSYM was calculated for the propulsive [KINSYM(+)], braking [KINSYM(-)], and net [KINSYM(net)] impulses generated by each limb as the ratio of the paretic impulse to the sum of the impulses generated by both limbs. Values were expressed as percent, with 50% representing perfect between-limb symmetry.

After familiarization outside the scanning environment, all subjects were asked to perform pedaling and foot tapping during fMRI. The purpose of tapping was to identify regions of the brain activated by leg movement. Our prior work in young, healthy controls showed that pedaling and tapping produced brain activation in similar cortical locations (Mehta et al., 2009). Hence, we reasoned that similar regions of activation during pedaling and tapping in stroke survivors would suggest that recorded signals were due to brain activation, not motion artifact.

**FIGURE 1:**
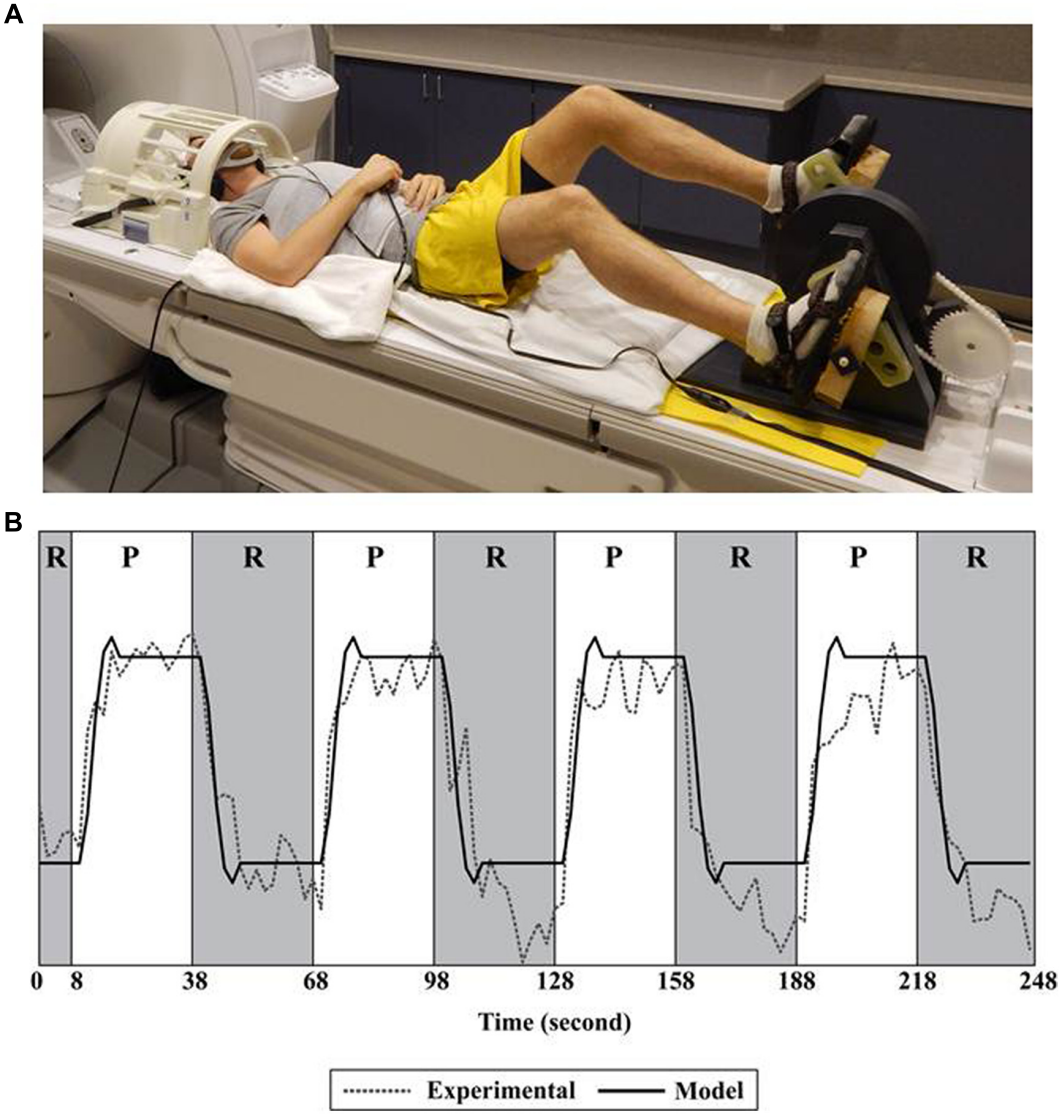
**Set-up and fMRI processing. (A)** Subject positioned for pedaling during functional magnetic resonance imaging (fMRI). **(B)** A single run consisted of 30 s of pedaling followed by 30 s of rest, repeated four times. The pedaling portion of the run (P) is shown in white; rest (R) is shown in gray. Recorded data (Experimental) were fit to a canonical function (Model) only during the rest portion of the run. Recorded data (Experimental) represent the time series of a single voxel in the cortex. See Mehta et al. (2009, 2012) for details.

During pedaling, the feet were fastened to a custom-designed device positioned on a scanner bed. See **Figure 1A**. This device has been described and validated for fMRI previously (Mehta et al., 2009). In brief, it was a direct-drive, flywheel-equipped apparatus constructed of non-metallic materials that provided a light mechanical workload (<2 J). The device was instrumented with an MRI-compatible rotary optical encoder (model: TD 5207, Micronor Inc., Newbury Park, CA, USA) that was coupled to the crank shaft. The encoder measured crank position from which pedaling rate was computed. Subjects were asked to pedal at a comfortable rate. We utilized a block design consisting of six runs of pedaling. As shown in **Figure 1B**, a single run consisted of 30 s of pedaling, followed by 30 s of rest, repeated four times. The duration of the blocks provided ample time for subjects to respond to the pedal cue, initiate leg moment, and achieve a steady pedaling rate, with time remaining for the blood-oxygen-level dependent (BOLD) signal to reach its peak. Similarly, there was adequate time to respond to the rest cue, terminate leg movement, and allow the BOLD signal to return to baseline (Malonek and Grinvald, 1996). Each run was preceded by 18 s of rest, 10 s of which were discarded to eliminate non-steady state magnetization artifacts.

During tapping, subjects’ legs were positioned over a foam bolster such that the hip and knees were flexed and the feet were approximately 15 cm above the surface of the scanner bed. A circular button (6.35 cm diameter) connected to a switch (Jelly Bean Twist Top Switch, AbleNet, Inc., Roseville, MN, USA) was placed under the foot. Subjects were asked to dorsi- and plantarflex the ankle at a comfortable rate to tap the button. Knee flexion/extension was allowed if ankle movement was not possible. Tapping was performed with one limb at a time. Each time the button was tapped, a pulse was generated. These data were used to calculate tapping rate. An event-related design consisting of three runs was used. A single run included 20 moving events and 74 resting events with 2 s per event, presented in random order. The parameters of the event-related design, including the number of events and the ratio of tap to rest events, were created with the *RSFgen* subroutine in Analysis of Functional NeuroImages (AFNI) software (Cox, 1996). The *Nodata* subroutine, also in AFNI, was used to test the adequacy of these parameters. Specifically, *NoData* used deconvolution to evaluate the shape of the hemodynamic responses created by the generated model.

During pedaling and tapping, a static tone indicated when to move; silence indicated rest. As shown in **Figure 1A**, the head was placed in a radio frequency coil and secured with a beaded vacuum pillow, a chin strap, and other padding as needed to minimize motion. The trunk was secured with a Velcro strap. Audio cues were delivered through MRI compatible ear buds. An additional set of headphones was used to protect against scanner noise. An emergency squeeze ball was provided. Participants were observed for safety and comfort and were able to communicate via intercom. We also had access to real time head position information. If we observed excessive head motion, we repositioned the head with additional care to limit motion and then restarted data collection. Pedaling and tapping were performed on two different days and counterbalanced to minimize ordering effects.

A 3.0T MRI scanner and a single channel transmit/receive split head coil were used (General Electric Healthcare, Milwaukee, WI, USA). Functional images (T2^∗^-weighted) were acquired using echo echoplanar imaging [repetition time (TR): 2000 ms, echo time (TE): 25 ms, flip angle: 77°, 36 contiguous slices in the sagittal plane, 64 × 64 matrix, 4 mm slice thickness, and field of view (FOV): 240 mm]. The resolution of the images was 3.75 mm × 3.75 mm × 4 mm. Anatomical images (T1-weighted) were obtained with a spoiled GRASS pulse sequence approximately half way through scan sessions (TR: 9.6 ms, TE: 39 ms, flip angle: 12°, 256 × 244 matrix, resolution: 1 mm^3^, FOV: 240 mm, 148 slices in the sagittal plane, and NEX: 1).

### Processing and Analysis

Processing of fMRI signals was completed using AFNI software. Dicom files containing fMRI signals were converted into 3-dimensional images. Individual voxels were aligned to the same temporal origin within each TR. The first four TRs within each run were removed to eliminate non-steady state magnetization artifacts. Multiple runs were concatenated and registered to the functional scan obtained closest in time to the anatomical scan. To identify voxels containing pedaling-related brain activation, general linear modeling was used to fit a canonical hemodynamic response function (a box car function convolved with a gamma function) to the measured BOLD signal. Conventional fMRI signal processing for block designs in which the entire BOLD signal is fit to a canonical function may not appropriate for measuring pedaling-related brain activation because limb and head motion can cause signal artifact (Mehta et al., 2009). To overcome this confound, we fit only the portion of the BOLD time-series after pedaling stopped (i.e., the rest period) to the canonical function. See **Figure 1B**. To do so, the canonical function was created “as if” all of the data (pedal and rest) would be used in analysis. Then, prior to fitting the data to the canonical function, we used the *censor* subroutine in AFNI to specify which time points would be included in analysis. The parameters of the *censor* subroutine were set to include the rest portion of the task and to exclude the pedaling portion. A total of 720 s of data (30 s per block, four blocks per run, six runs) were included in analysis. This approach has been validated previously (Mehta et al., 2009) and is justified because the onset and termination of BOLD signals are delayed with respect to behavior (Blamire et al., 1992; Bandettini and Cox, 2000). Hence, movement-free BOLD signal remains present after pedaling has stopped. Based on known hemodynamic responses to local neuronal activity (Blamire et al., 1992; Malonek and Grinvald, 1996; Bandettini and Cox, 2000), approximately 1/3 (i.e., 10 s) of the signal analyzed represented pedaling-related brain activation. The remainder represented rest. To identify voxels containing tapping-related brain activation, voxel-wise hemodynamic response functions were used. Head position was used as a variable of no interest. Functional data were blurred using a full width half maximum Gaussian filter. To identify significantly active voxels at a family wise error rate of *p* < 0.05, we used Monte Carlo simulation (AlphaSim) to set an appropriate cluster size for a given individual voxel *p*-value. Cluster sizes were determined individually for each subject, and depending on the subject, were 7–8 voxels. The input parameters to AlphaSim were the subject’s whole brain image, the size of the Gaussian filter, the cluster connection radius, the individual voxel probability, and the number of iterations. Cluster connection radius (6.6 mm), individual voxel probability (0.005), and number of iterations (1000) remained constant across all subjects. Gaussian filter parameters varied across subjects because the blur level input was based on the inherent blur value in each subject’s data. We used the subroutine *3dFWHMx* in AFNI to compute the blur level. Significantly correlated voxels outside of the brain and negatively correlated voxels were ignored. Voxels with percent signal change greater than 10 were also ignored, as these large changes were likely due to edge effects.

Quantitative measures of brain activation were extracted from each subject’s data individually, in their native coordinate system. The decision to analyze the data in native space (not standard space) was based on the observation that, in some stroke subjects, the lesion eliminated anatomical landmarks used for standardization. This was a concern, as absence of landmarks could lead to errors in standardization and inaccurate localization of brain activation.

Measures were extracted from M1/S1, Brodmann’s area 6 (BA6), and cerebellum (vermis and lobules IV, V, and VIII). Anatomical boundaries for each region were defined from T_1_-weighted images in native space as previously described (Wexler et al., 1997; Schmahmann et al., 1999; Jenkins et al., 2000). Quantitative measures were extracted from each region and from all regions combined. Measures included volume, intensity, and laterality of activation. Volume was defined as the number of significantly active voxels in each activated region multiplied by voxel volume in microliters (μL). Intensity was defined as the average percent signal change from baseline in the active portion of the region of interest. Laterality of activation was measured by LI, defined as the difference in volume between the damaged and undamaged sides (stroke) or left and right sides (control) as a proportion of total volume on both sides of the brain.

After finding no significant effects of lesion location (cortical vs. subcortical), group means (SD) for volume, intensity, and LI were computed for stroke and control subjects. Multivariate general linear modeling (alpha = 0.05) was used to test for between-group differences (stroke vs. control) in volume, intensity, and LI. Measures were computed for each active region and for all active regions combined.

The decision to extract measures from M1/S1, BA6, and cerebellum was based on visual inspection of brain activation maps on a subject-by-subject basis. This decision was further supported by inspection of group activation maps and prior knowledge from control subjects about regions normally involved in pedaling. In examining the data in a subject-by-subject basis in native space, we consistently saw clear clusters of activation in M1, S1, BA6, and cerebellum. While some subjects also had smaller, scattered activations outside these regions, no other region was consistently active, across subjects, within groups. Hence, we concluded that these were the only regions that we could confidently say were involved in pedaling. Hence, they were selected for analysis. However, we were also concerned that identifying regions in this manner might have resulted in under-detection of meaningful activation in the stroke group. Thus, we also decided to average the data and test for significant clusters of activation outside M1/S1, BA6, and cerebellum. To this end, data were transformed into the standardized coordinate system of Talairach and Tournoux (1988), blurred, and averaged across subjects to obtain mean activation maps for each group and each condition. To achieve standardization, we manually estimated the location of the landmarks. Here, we were less concerned about the accuracy of localization. We reasoned that if a cluster(s) outside of M1/S1, BA6, or cerebellum reached significance, we could go back into native space to more precisely localize the active region. Our purpose, at this point, was to determine whether there was any pattern to the seemingly inconsistent activations that we saw in individual subjects. After group averaging, there were no significant clusters of activation outside M1/S1, BA6, and cerebellum. This knowledge provided further confidence that we were not overlooking substantial activations outside the regions selected for analysis.

The decision to quantify activation only in brain regions consistently active across subjects meant that we may have overlooked small, task-associated activations unique to individuals. However, it also prevented us from over-interpreting small and inconsistent activations. Moreover, this approach enhanced our ability to make inferences about stroke survivors as a whole because activations in the brain regions evaluated were consistent across subjects. Finally, this approach allowed us to compare brain activation across groups in regions normally involved in pedaling. Indeed, our prior work in young, healthy controls revealed significant pedaling-related brain activation that was limited to M1, S1, supplemental motor area, and cerebellum (Mehta et al., 2009, 2012).

Head motion was measured indirectly from volume registration calculations performed in AFNI and was characterized in terms of displacement, oscillation, and drift. Displacement was defined as the mean distance between the position of the head and the registration point. Oscillation was defined as the mean variation (i.e., SD) in head position about the registration point. Drift was the absolute distance between the position of the head at the start and end of a condition. Values for head motion were computed in the *x* (medial/lateral), *y* (anterior/posterior), and *z* (inferior/superior) direction for each subject and each condition. Independent *t*-tests (alpha = 0.05) were used to examine between-group differences in each type of head movement, in each direction.

Several *post hoc*, exploratory analyses were conducted to better understand between-group differences in pedaling-related brain activation. Pearson correlation coefficients were used to examine the relationship between brain activation volume and head motion, pedaling rate, and the impairment measures reported in **Table 1** [i.e., FM_LEtotal_, FM_LEmotor_, FM_LEsens_, %Work(+), %Work(-), %Work(net), TSR, SLR, KINSYM(+), KINSYM(-), KINSYM(net)]. We also compared tapping rate and volume among control, paretic, and non-paretic limbs. As with the pedaling data, multivariate general linear modeling was used (alpha = 0.05) to compare separate regions of activation and all regions combined.

## Results

### Task Performance

**FIGURE 2:**
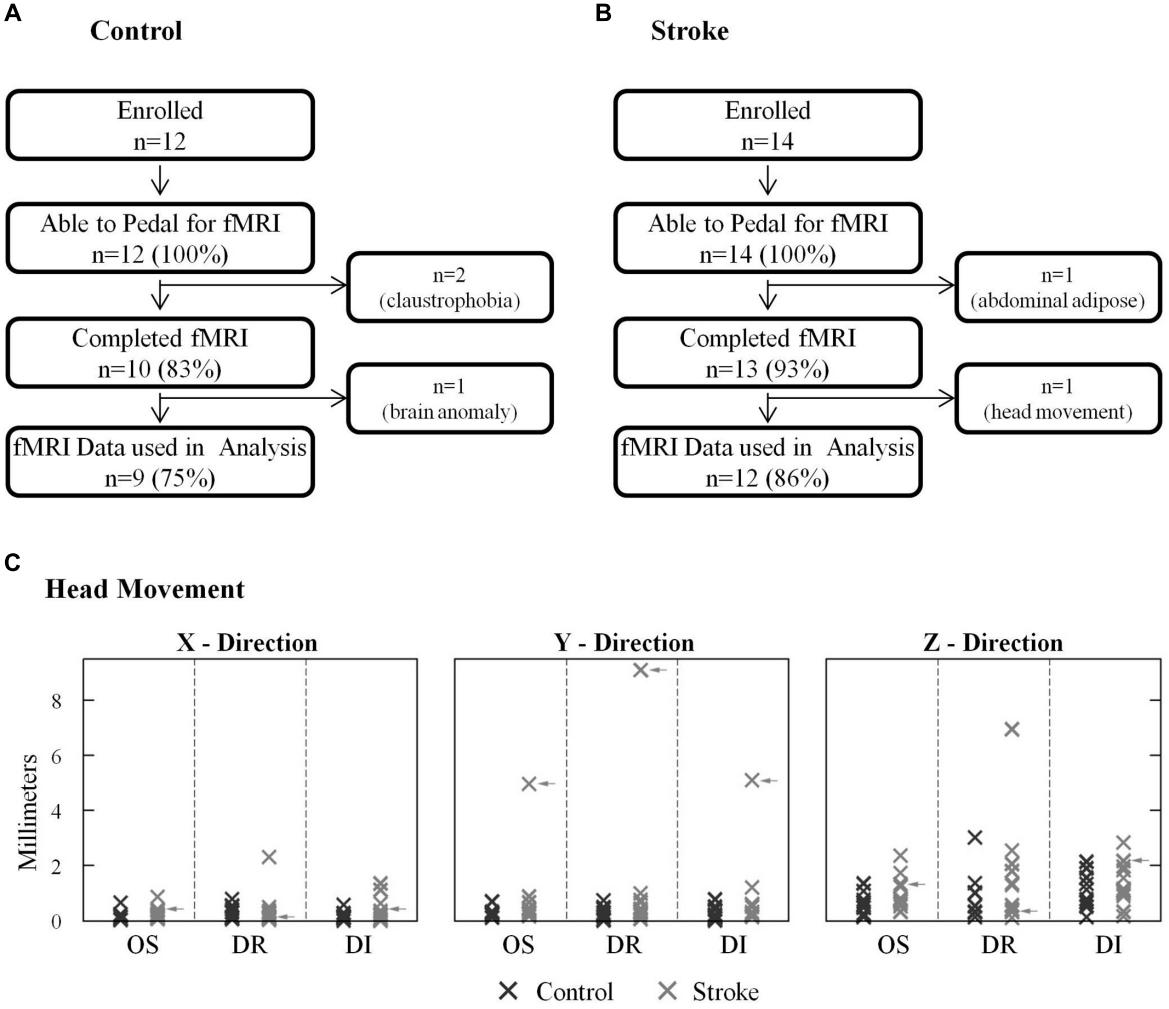
**Successful task completion and head motion. (A,B)** All stroke and age-matched control subjects were capable of pedaling the custom device with their head and body positioned for fMRI. Two control subjects could not complete fMRI due to claustrophobia, and one stroke subject could not enter the gantry due to abdominal adiposity. One control subject’s fMRI data could not be used due to an incidental finding. fMRI data from 1 stroke subject was not used in analysis due to excessive head motion. **(C)** Head motion was similar in stroke and control groups. In most subjects head motion was <3 mm. Data from the subject who was excluded due to head motion are indicated with arrows.

All stroke and age-matched control subjects could pedal the custom device with the head properly positioned in the radio frequency coil and the torso restrained, as required for fMRI (**Figures 2A,B**). All but three subjects completed all the experimental procedures. There we two other subjects whose fMRI data were not used in analysis. Hence, 81% of subjects enrolled provided a complete data set. With respect to data loss, two control subjects were withdrawn due to claustrophobia. One stroke subject was unable to enter the gantry due to excessive abdominal adipose tissue. One control subject’s data were excluded from analysis due to an incidental finding. fMRI data from one stroke subject, who completed all procedures, were not used in analysis because physiologically plausible, pedaling-related brain activation was not detected. In this subject, instead of seeing clusters of brain activation localized to particular regions of gray matter, we observed a small number of significant voxels that were scattered, in a non-localized fashion, across the entire brain (i.e., gray matter, white matter, and ventricles). Upon examination of head motion data, we learned that this subject had ≥5 mm of oscillation and displacement and >9 mm of drift in the *y* direction (**Figure 2C**). Head motion in most subjects did not exceed 3 mm, and no other subject displayed such extensive head motion. Hence, the absence of physiologically plausible fMRI signal was attributed to excessive head motion, and fMRI data were not included in analysis. After removing this subject’s data, there were no significant differences (*p* > 0.07) between control and stroke groups for any type of head motion in any direction. There was also no significant between-group difference in pedaling rate [*p* = 0.14, control = 0.95 (0.18) Hz, stroke = 0.81 (0.23) Hz].

### Brain Activation

**FIGURE 3:**
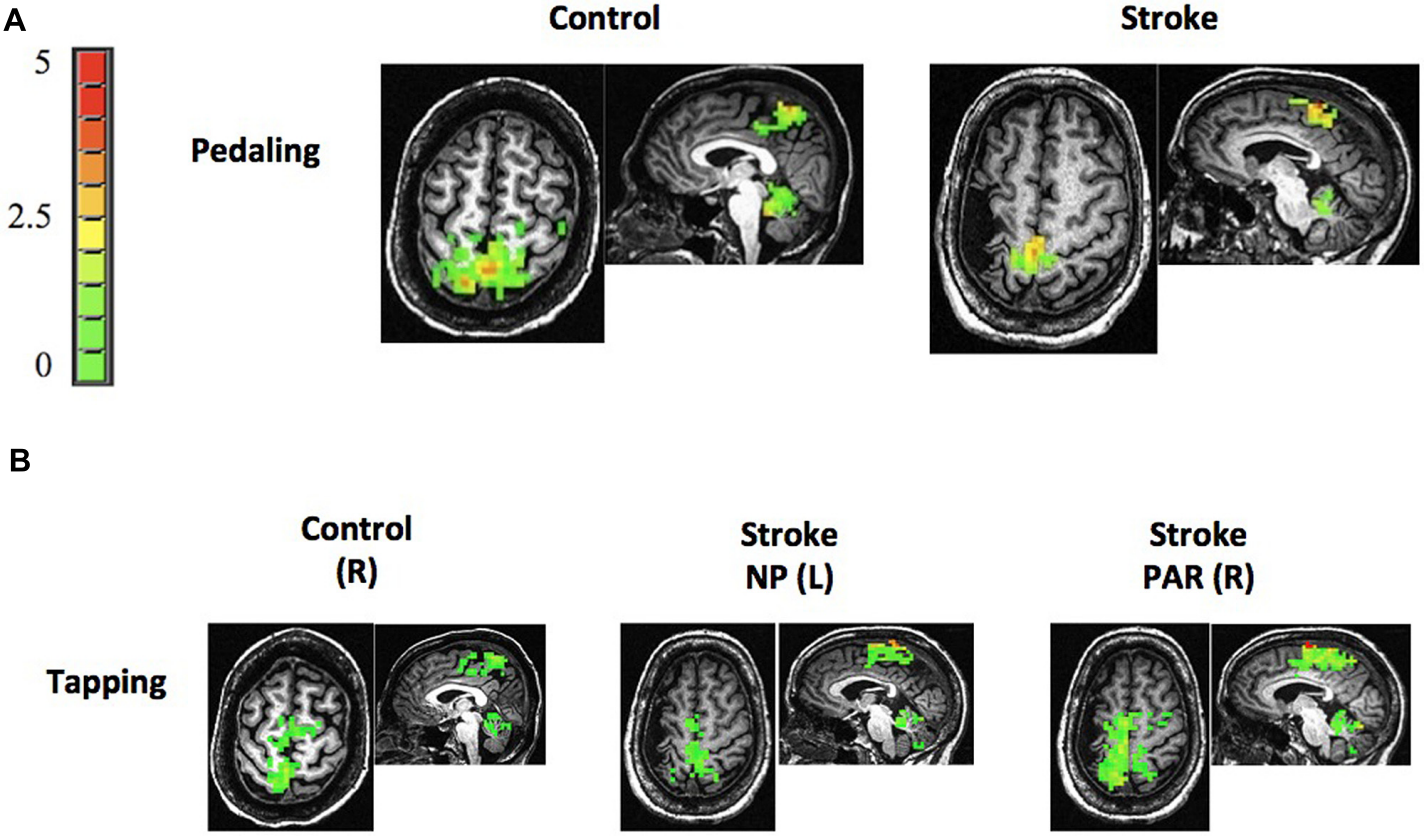
**Brain activation. (A,B)** Representative examples of brain activation associated with pedaling and tapping in control and stroke subjects. Both groups displayed pedaling-related brain activation on both sides of M1, S1, Brodmann’s area 6 (BA6), and cerebellum. Note that brain activation associated with pedaling was spatially consistent with that seen during tapping and with leg areas of the cortex. The color bar represents percent signal change (0–5%). R, right; L, left; NP, non-paretic; P, paretic.

Stroke and age-matched control subjects displayed pedaling-related brain activation in M1, S1, BA6, and cerebellum (vermis and lobules IV, V, and VIII; **Figure 3**). In both groups, brain activation was apparent in the medial aspect of M1 and S1, consistent with the leg area of the sensorimotor cortex. Moreover, the location of cortical activation associated with pedaling was consistent with that observed during lower limb tapping (Compare **Figures 3A,B**). Pedaling-related brain activation was apparent on both sides of the cortex and cerebellum, as would be expected for a bilateral movement. Values for LI were -0.06 (0.20) in the stroke cortex, 0.05 (0.06) in the control cortex, 0.29 (0.33) in the stroke cerebellum, and 0.04 (0.15) in the control cerebellum. In the cortex, there was no significant between-group difference in LI (*p* = 0.10), and LI was not significantly different from zero in the stroke group (*p* = 0.34). In the cerebellum, LI was significantly different between groups (*p* = 0.03), and within the stroke group, LI was significantly different from zero (*p* = 0.01). Hence, activation in the cerebellum, but not the cortex, was significantly lateralized toward the lesioned side of the brain. Brain activation maps were free from edge effects, activation in the ventricles, and other characteristics of motion artifact (Kruger and Glover, 2001; Huettel et al., 2004).

**FIGURE 4:**
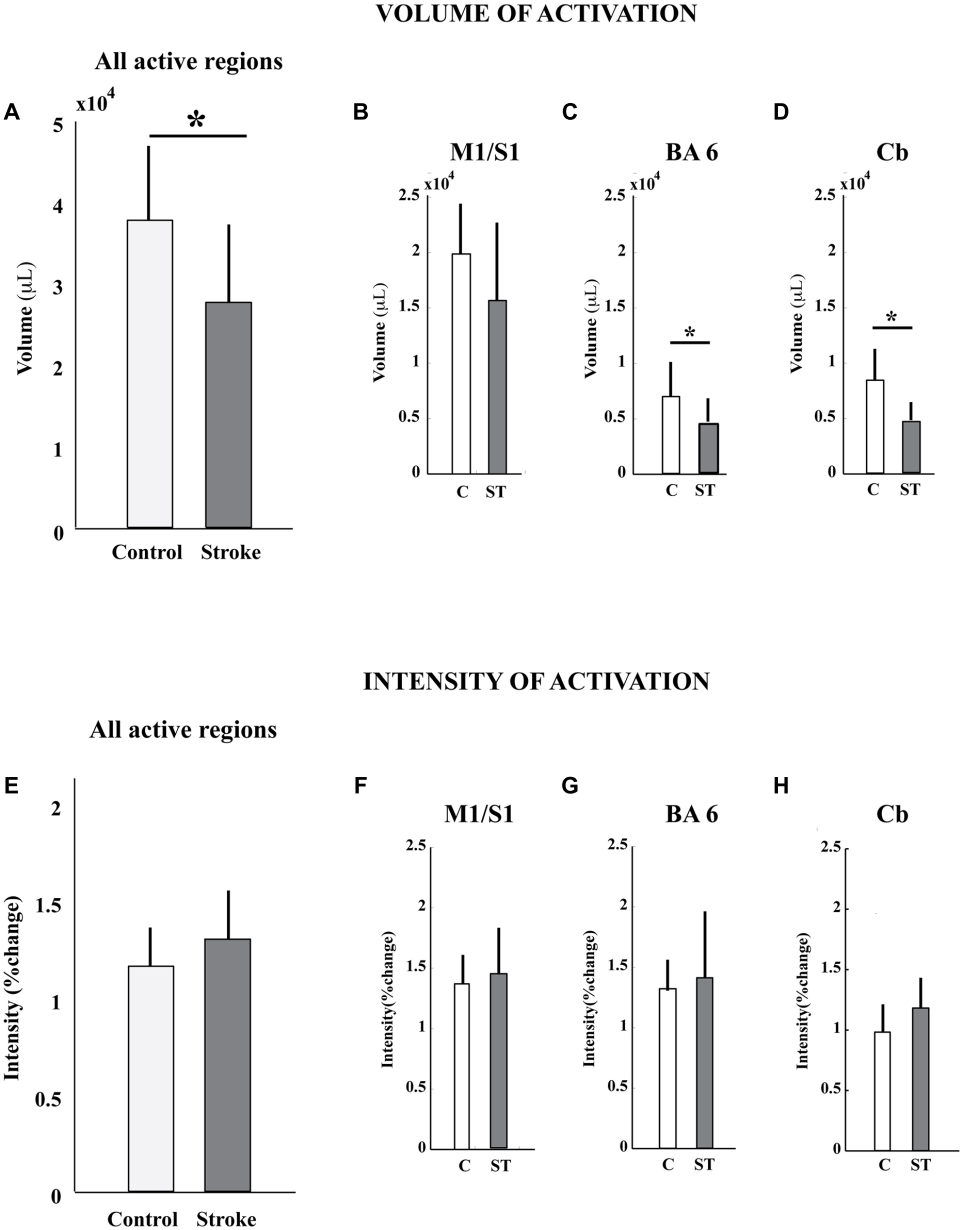
**Volume and intensity of pedaling-related brain activation. (A–D)** The volume of pedaling-related brain activation was reduced in stroke subjects, as compared to control. Differences reached statistical significance in all active regions combined, Brodmann’s area 6, and cerebellum. **(E–H)** The intensity of brain activation associated with pedaling was not significantly different between groups. Values are group mean (SD). Asterisks indicate significance at *p* < 0.05. C, control; ST, stroke; BA6, Brodmann’s area 6; Cb, cerebellum.

The volume, but not the intensity, of pedaling-related brain activation was different between stroke and control groups (**Figure 4**; **Table 2**). When all active regions were examined together, volume was significantly smaller in the stroke group as compared to control. When individual regions were examined separately, reduced brain activation volume reached statistical significance in BA6 and cerebellum, but not in M1/S1. Regardless of whether activated regions were examined together or separately, there were no significant between-group differences in brain activation intensity.

**Table 2:** Volume and intensity of pedaling-related brain activation as measured by fMRI.

Brain region	Group	Volume (μL)	Intensity (% signal change)
All active regions	Stroke	27,694 (9,608)	1.30 (0.25)
	Control	37,819 (9,169)	1.16 (0.20)
		*p* = 0.03	*p* = 0.17
M1/S1	Stroke	15,647 (7,038)	1.44 (0.38)
	Control	19,863 (4,543)	1.37 (0.24)
		*p* = 0.13	*p* = 0.64
Brodmann’s area 6 (BA6)	Stroke	4,350 (2,347)	1.41 (0.55)
	Control	6,938 (3,134)	1.32 (0.24)
		*p* = 0.04	*p* = 0.64
Cerebellum (vermis, IV, V, VIII)	Stroke	4,591 (1,757)	1.18 (0.25)
	Control	8,381 (2,835)	0.98 (0.23)
		*p* = 0.001	*p* = 0.70

*Values are mean (SD).*

### *Post hoc*, Exploratory Analysis of Reduced Volume

**FIGURE 5:**
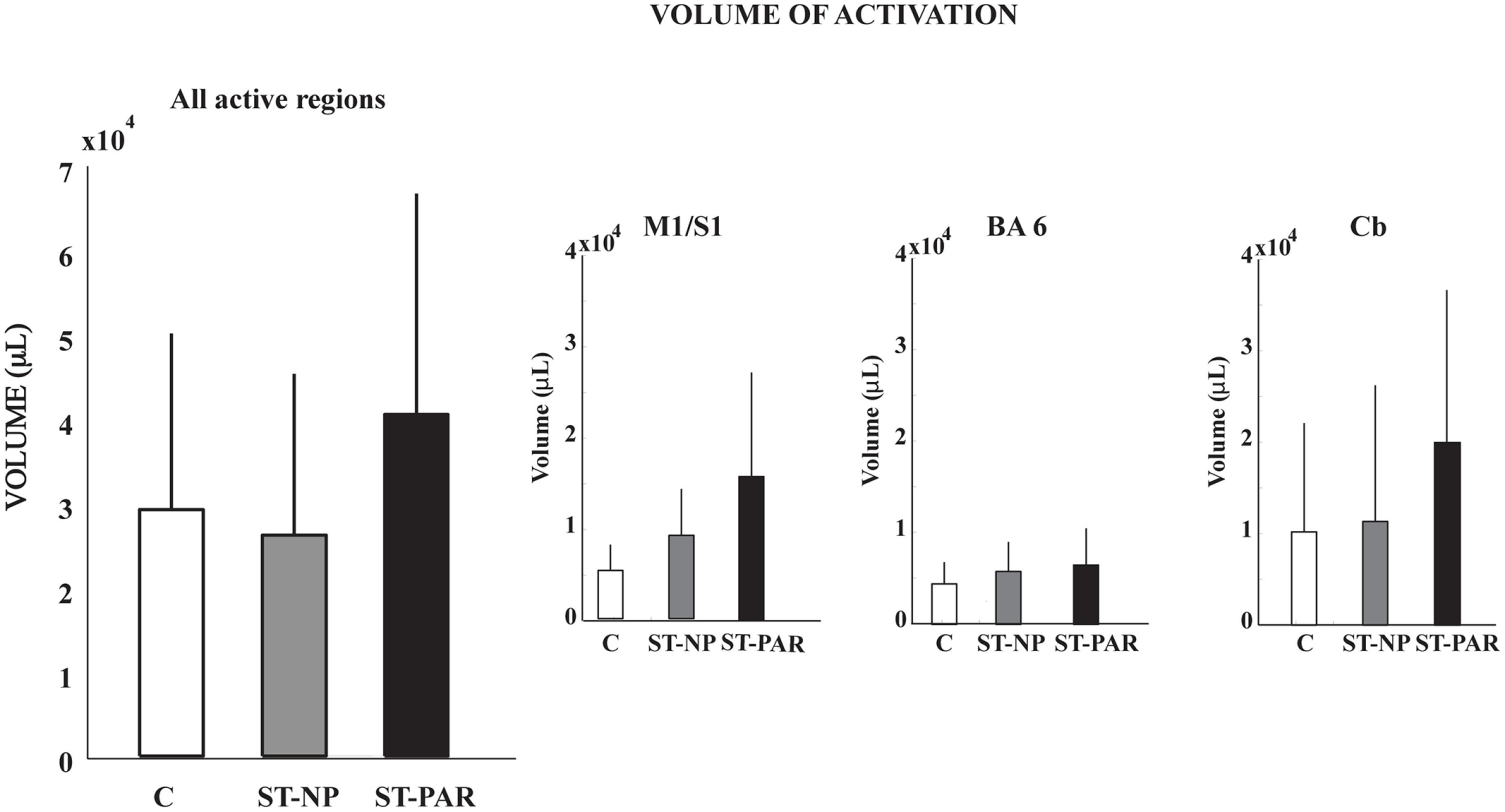
**Volume of tapping-related brain activation**. There was no difference in the volume of tapping-related brain activation for stroke and control subjects. C, control; ST, stroke; PAR, paretic; NP, non-paretic; BA6, Brodmann’s area 6; Cb,cerebellum.

Having observed reduced brain activation volume during pedaling in the stroke group, we performed several *post hoc*, exploratory analyses to better understand this result. First, we sought to understand whether head motion could have interfered with fMRI signal detection, leading to decreased volume. Thus, we examined the relationship between volume and head motion within the stroke group. Here, we found no significant correlation between volume and any type head motion, in any direction (**Table 3**). We also examined the relationship between volume and pedaling rate, as stroke subjects had a tendency to pedal more slowly than control. However, we found no significant correlation between pedaling rate and volume (*r* = 0.045, *p* = 0.895.) We also found that tapping rate for paretic limbs [1.37 (90.3) Hz] tended to be lower than for non-paretic [1.66 (0.36) Hz] and control limbs [1.87 (0.69) Hz] (*p* = 0.09). However, unlike pedaling, tapping-related brain activation volume was not significantly reduced in the stroke group, as compared to control or non-paretic limbs. Instead, there was a tendency for elevated volume during paretic tapping, as compared to non-paretic and control tapping that did not reach statistical significance, see **Figure 5** and **Table 4**. As shown in **Table 5**, there were no significant correlations between volume of brain activation and any measures of stroke-related impairment. The only measure that approached significance was %Work(net) (*r* = 0.525, *p* = 0.080). In other words, volume tended to increase with increasing work performed by the paretic limb during pedaling.

**Table 3:** Relationship between head motion and volume of pedaling-related brain activation.

	Direction of Motion			
Type of motion		x	y	z
Oscillation	*r*	0.30	0.22	0.10
	*p*	0.42	0.54	0.80
Drift	*r*	0.30	0.41	0.14
	*p*	0.42	0.26	0.70
Displacement	*r*	0.03	0.02	0.10
	*p*	0.93	0.95	0.41

*x, medial/lateral; y, anterior/posterior; z, inferior/superior.*

**Table 4:** Volume of tapping-related brain activation as measured by fMRI.

Brain region	Group	Volume (μL)
All active regions	Paretic	40,509.4 (26,238.3)
	Non-paretic	26,207.8 (19,112.3)
	Control	29,230.2 (20,902.4)
M1/S1	Paretic	15,703.1 (11,392.9)
	Non-paretic	9,093.8 (5,085.8)
	Control	5,253.1 (2,838.9)
BA6	Paretic	6,492.2 (4028.0)
	Non-paretic	5,779.7 (3243.8)
	Control	4,425.0 (2387.2)
Cerebellum (vermis, IV, V, VIII)	Paretic	19,979.0 (16,675.7)
	Non-paretic	11,334.4 (14,893.6)
	Control	10,200.0 (11,903.4)

*Values are mean (SD).*

## Discussion

The results of this study provide two original and important contributions. First, we demonstrated that pedaling can be used with fMRI to examine brain activation associated with lower limb movement in people with stroke and age-matched controls. Unlike many other lower limb movements studied with fMRI (Carey et al., 2004; Dobkin et al., 2004; Luft et al., 2004, 2005; Jang et al., 2005; You et al., 2005; Kim et al., 2006; Enzinger et al., 2008, 2009; MacIntosh et al., 2008), pedaling involves continuous, reciprocal, multijoint movement of both lower limbs. In this respect, pedaling has many characteristics of functional lower limb movements, such as walking. Thus, the importance of our contribution lies in the establishment of a novel paradigm that can be used to understand how the brain adapts to stroke, recovery, and rehabilitation in order to produce functional lower limb movements. Second, we found that brain activation volume during pedaling was reduced in people post-stroke, as compared to age-matched controls. Reduced volume could not be fully explained by differences in pedaling rate or head motion, but was associated with work produced by the paretic leg during pedaling. Reduced brain activation volume may be due to anatomic, physiology, and/or behavioral changes after stroke, but methodological issues cannot be excluded. Importantly, brain activation volume post-stroke was both task-dependent and mutable, which suggests that it could be modified through rehabilitation.

### Recording Pedaling-Related Brain Activation in Stroke and Age-Matched Controls

Consistent with our primary aim, this study demonstrated that brain activation could be recorded with fMRI during pedaling in individuals with stroke and age-matched controls. When we began this work, we were concerned that abnormal posture associated with hemiparesis or advanced age could make it difficult to position the head and torso for fMRI. We also considered that stroke survivors might have difficulty pedaling our device. Hyperreflexia, abnormal synergies, or jerky pedaling movements could have caused excessive head motion. Our concerns were not substantiated. All stroke and age-matched controls could pedal, as instructed, while supine on the scanner bed with the head positioned in a radio frequency coil. Moreover, head motion was successfully minimized in all but one subject. In 22 out of 23 subjects who pedaled during fMRI, head motion was not markedly larger than that observed in prior work with young, healthy adults (Mehta et al., 2009, 2012). Moreover, brain activation maps were free from edge effects indicative of motion artifact (Kruger and Glover, 2001; Huettel et al., 2004).

Several technical and human factors contributed to successful head stabilization. The beaded vacuum pillow and chin strap made it difficult to move the head more than a few millimeters. During familiarization, we stressed the importance of controlling head motion. We encouraged subjects to work with us to achieve a comfortable and secure head position that could be maintained for the entire scan. We scheduled ample time for set-up, therefore allowing time to repeatedly reposition the head until stabilization was achieved. In short, the study team and our subjects were acutely aware of the importance of stabilizing the head; so great care and cooperation were exercised before scanning.

Despite excellent head stabilization, some motion during pedaling was unavoidable. Such motion was task-correlated and therefore could have been problematic, as task-correlated motion can cause erroneous signal detection (Hajnal et al., 1994; Field et al., 2000; Arnold et al., 2003). Hence, another important factor in addressing motion was our fMRI signal processing technique. Recall, we extracted the portion of the BOLD time-series after pedaling stopped (i.e., the rest period) and fit only this portion of the signal to a canonical function. This approach was justified because head motion was minimal at rest; yet the onset and termination of BOLD signals are delayed with respect to behavior (Blamire et al., 1992; Bandettini and Cox, 2000). Therefore, motion-free, task-associated BOLD signal is present after pedaling stops. We validated this method in a prior publication (Mehta et al., 2009).

While motion artifact was not a major impediment to recording pedaling-related brain activation with fMRI, we did discard one data set due to excessive head motion. It is unclear why head motion could not be adequately controlled in this case, as there was nothing atypical about the subject or the circumstances. Possibilities include individual variation in anatomy, posture, or behavior, limitations in equipment for head stabilization, and/or human error on the part of the study team. The loss of one data set due to head motion also demonstrates that there are limitations to signal processing techniques for minimizing motion artifact. Specifically, our technique whereby we processed only the BOLD signal acquired during rest was not effective when head motion reached ∼9 mm. It is possible that even smaller amounts of head motion preclude valid data. However, our data do not allow us to pinpoint a maximal allowable value for head motion. Thus, care should be taken to limit head motion as much as possible, even when processing only the rest period of the data.

There were four other cases in which claustrophobia, body composition, or incidental findings interfered with data collection or analysis. Important to our aims, failure to acquire or use these data was unrelated to pedaling *per se*, pedaling during fMRI, or head motion. All four of these subjects had a successful familiarization, passed the fMRI screening, and pedaled the custom device with the head positioned in the radio frequency coil. Individuals who experienced claustrophobia reported no prior problems with small or enclosed spaces, but also had no prior experience with MRI. This observation suggests that, while necessary and important, screening is not a perfect predictor of comfort with MRI. Similarly, the subject whose body type prohibited data collection had a body weight within acceptable limits for MRI. However, because the base of our pedaling device sits on top of the scanner bed and subjects lie on the base, space within the gantry is reduced from normal. With this subject, the combination of reduced space and large body size prevented his abdomen from entering the gantry. Consequently, our screening now considers body weight as well as distribution of body fat with respect to the reduced space in the gantry. Finally, data loss due to incidental findings was below expected rates. Prior work has demonstrated that incidental findings are detected in 18% of asymptomatic volunteers undergoing brain MRI (Katzman et al., 1999).

In summary, our observations demonstrate that it is feasible to record pedaling-related brain activation with fMRI in people with stroke and age-matched controls. However, some data loss can be expected due to motion artifact and other known obstacles to fMRI, such as claustrophobia, obesity, and incidental findings. Recruitment and enrollment efforts for similar studies should account for approximately 20% data loss. However, we also acknowledge that our sample was limited to community dwelling, chronic stroke survivors. Data loss could be higher, if more impaired stroke survivors are recruited and enrolled.

### Reduced Brain Activation Volume Post-Stroke

The second major finding of this study was that, in comparison to the control group, people post-stroke displayed reduced volume in brain regions activated by pedaling. Reduced volume reached statistical significance in all active regions except M1/S1. However, even M1/S1 displayed a tendency for reduced volume, and this effect may have reached statistical significance in a larger sample. Hence, we conclude that reduced activation volume during pedaling was not unique to a particular region(s). Rather, reduced volume appears to be a generalized finding across brain regions activated by pedaling. We have considered a number of methodological, anatomical, physiological, and behavioral explanations for this observation. Each is discussed below.

With respect to methodological factors contributing to decreased brain activation volume during pedaling post-stroke, we considered that our imaging method; i.e., BOLD-fMRI, may have underestimated brain activation in the stroke group. The spatiotemporal characteristics of hemodynamic responses can be abnormal after stroke, and these abnormalities may result in underrepresentation of brain activation as measured by fMRI (Newton et al., 2002; Pineiro et al., 2002; Fridriksson et al., 2006; Roc et al., 2006; Bonakdarpour et al., 2007; Altamura et al., 2009; Mazzetto-Betti et al., 2010). To ensure that this methodological issue did not confound our results, the present study was preceded by an examination of the spatiotemporal profile of the hemodynamic responses of each stroke survivor enrolled (Promjunyakul et al., 2013). This investigation revealed only small changes in the size and shape of hemodynamic responses. Changes were not as substantial as other reports and were not large enough to produce inaccurate brain activation maps. Hence, underrepresentation of brain activation due to BOLD-fMRI is an unlikely explanation for reduced volume.

It is also possible that decreased pedaling-related brain activation volume in the stroke group was due to inadequate power to detect active voxels. Recall that we fit only the rest portion of the data to a canonical function, thereby reducing the number of TRs used for analysis to half of the number collected. While possible, several lines of reasoning suggest that this explanation is unlikely and that the number of TRs was adequate for signal detection. In two prior studies of young, healthy controls, we used the same fMRI signal processing technique described here (Mehta et al., 2009, 2012). In those studies, 180 TRs were included in analysis (15 TRs per block, four blocks, three runs), which was adequate for or detecting pedaling-related brain activation. However, we were concerned that fMRI signals could be more difficult to detect in older adults and people with stroke. Hence, for this study, we doubled the number of pedaling runs performed by both groups (i.e., we used six runs instead of three), which provided 360 TRs. Doubling the number of TRs did not change the brain activation maps in controls. So, in the healthy brain, we are confident that we have adequate power to detect brain activation. Nevertheless, the question remains as to whether the number of TRs was adequate to detect brain activation in stroke subjects. Several pieces of evidence suggest that it was. First, we analyzed the same number of TRs in stroke and control subjects. Thus, from a sampling perspective, both groups had the same opportunity to display brain activation. Therefore, if power were compromised in the stroke group, this problem would have had to arise from other methodological factors that reduce the magnitude of task-related signal change (e.g., abnormal hemodynamic responses) or inflate noise (e.g., excessive head motion). However, hemodynamic responses were not blunted in the stroke group (Promjunyakul et al., 2013). Moreover, head motion was similar in the two groups and was not correlated with volume. Thus, from a methodological perspective, it does not appear that signals were more difficult to detect in stroke as compared to control subjects, suggesting that inadequate power cannot easily explain reduced volume post-stroke.

Another methodological factor that we explored was pedaling rate. In this study, stroke subjects pedaled ∼15% more slowly than age-matched controls. Indeed, fMRI signals often scale with movement rate (Rao et al., 1996; Sadato et al., 1997; Jancke et al., 1999; Khushu et al., 2001). Therefore, slower pedaling rate could explain reduced volume. However, if this were the case, one would expect a positive correlation between rate and volume. No such correlation was observed. The tapping data provide further evidence that movement rate is not a good explanation for our results. As with pedaling, tapping rate was slower in stroke as compared to control subjects. However, tapping produced no difference in brain activation volume in stroke as compared to control subjects. While these observations fail to provide compelling evidence that pedaling rate accounted for reduced volume, it is possible that the *post hoc*, exploratory analyses lacked adequate power to detect significant effects. Indeed, this study was not designed to examine these hypotheses, and future work should consider these issues explicitly. For example, the rate question could be addressed by systematically manipulating pedaling rate or by examining brain activation at fixed rates across subjects and groups. Moreover, it would be helpful to understand how the observed movement rates compared to subjects’ maximal ability. Here, all subjects were asked to pedal at a comfortable rate. The intent of this instruction was to match effort and/or difficulty. However, self-selected comfortable rate may be more difficult for some subjects than others. For example, control subjects might have pedaled at a rate closer to their maximal ability as compared to stroke. If this were the case, then between-group differences in volume could be due to differences in task difficulty or effort. Future work should also compare movement rate to maximal rate to address this issue.

Regarding anatomical contributions to our observations, we considered that reduced pedaling-related brain activation volume post-stroke may have been caused by a reduction in viable brain tissue. Indeed, all stroke survivors suffered some tissue loss. However, this explanation is unlikely because no stroke survivors had lesions affecting the medial aspect of the pre- and post-central gyri where the leg areas of M1 and S1 are located. Moreover, no subjects had lesions in the cerebellum. Thus, brain activation was lacking in apparently vital regions that are typically involved in pedaling. The idea that the stroke-affected brain failed to engage, during pedaling, portions of the brain that are capable of activity is further supported by the observation that the volume of activation across all active regions was larger during tapping (∼40,000 μL) as compared to pedaling (∼30,000 μL), suggesting that tissue capable of activation failed to display activity during pedaling. This observation further suggests that brain activation post-stroke is mutable and task-dependent; i.e., it can be elicited by some behaviors and not others.

Reduced activation volume in anatomically intact brain regions could be explained by impaired structural connectivity among regions due to white matter damage. Seven of the stroke survivors examined had subcortical lesions involving the internal capsule, corona radiata, basal ganglia, or thalamus. Subjects who were classified as cortical strokes also displayed some white matter disruption. Loss of white matter could render ineffective signals from intact portions of the cerebral cortex and cerebellum. Unless these signals find alternative pathways, brain regions may cease to fire for lack of meaningful effects on intended targets. Similarly, loss of structural connectivity could reduce sensory input reaching the cortex, which is a major source of pedaling-related cortical activation (Christensen et al., 2000; Mehta et al., 2012). While plausible, these hypotheses require further study because evidence is lacking. Hamzei et al. (2006, 2008) have shown that stroke survivors with substantial corticospinal tract lesions affecting gray or white matter displayed *increased* movement-related brain activation volume and intensity after training; whereas, stroke survivors with an intact corticospinal tract showed *decreased* M1 and S1 activity with training and recovery. This group suggested that the lack of effective connections between motor areas of the brain and their targets led to compensatory over-activity, not under-activity, of cortical tissue.

With respect to a physiological explanation, we considered that exaggerated cortical inhibition could contribute to reduced pedaling-related brain activation volume post-stroke. Prior work suggests that exaggerated interhemispheric inhibition due to stroke reduces cortical output through transcallosal effects exerted over M1 in the damaged hemisphere (Traversa et al., 1998; Murase et al., 2004). However, this phenomenon manifests as lateralized activation of the hemispheres such that the damaged sensory and motor cortices are less active than the undamaged cortices. In the present study, LI revealed no significant difference in the volume of cortical activation on the left and right side. Thus, if interhemispheric inhibition contributed to our observations, it did so in conjunction with other mechanisms such that the combined effect produced the same volume of activation in the left and right cortices.

Also with respect to interhemispheric inhibition, another novel finding of this study was that people post-stroke displayed asymmetric activation in the cerebellum during pedal that was characterized by a greater volume on the damaged side of the brain as compared to the undamaged side. In the control group, the volume of cerebellar activation was the same on the left and right sides. To our knowledge, this is the first description of cerebellar activity post-stroke during a continuous, reciprocal, multijoint movement of both lower limbs. Hence, there are no observations to which our data can be compared, and we made no *a priori* predictions about stroke-related changes in cerebellar activity during pedaling. However, having observed this phenomenon, we suggest the following interpretation. Ipsilesional cerebellar activation may compensate for unopposed interhemispheric inhibition from the undamaged to the damaged cortex and may help restore symmetrical activation across cerebral hemispheres. Ugawa et al. (1991) have demonstrated that transcranial electrical stimulation of the human cerebellum can disfacilitate the contralateral motor cortex. The structures likely to be responsible for this phenomenon are the dento-thalamic-cortical and the cerebellar-thalamic-cortical pathways, arising from the dentate and interpositus nuclei of the cerebellum, respectively. The regions of the cerebellum that were active during pedaling – lobules IV, V, and VIII – send output to these nuclei (Kandel et al., 1991). These observations suggest that the cerebellar activity observed during pedaling may have an inhibitory effect on the cerebral cortices. Cerebellar activity was more substantial on the damaged side of the brain as compared to the undamaged side. Consequently, cortical inhibition arising from the cerebellum would have a more substantial effect on the undamaged motor cortex as compared to the damaged side. Hence, imbalanced cerebellar activity might be a compensatory mechanism to minimize unopposed interhemispheric inhibition from the damaged to the undamaged hemisphere and to restore balanced activity between the two hemispheres. Consequently, cerebellar activity may help explain why sensory and motor cortex activity on the left and right side of the brain was not different in volume or intensity during pedaling post-stroke.

It is also possible that reduced pedaling-related brain activation volume post-stroke is due to enhanced reliance on spinal and/or brainstem structures for lower limb movement. It is well established that, in non-human animals, the spinal cord can produce the basic pattern of rhythmic, reciprocal, multi-joint muscle activity that characterizes locomotion (Duysens and Van de Crommert, 1998). The brainstem also contains pattern generating circuits (Le Ray et al., 2011). Evidence for spinal and brainstem locomotor generators in human is less convincing than in non-human animal, and it is thought that cortical signals are required to initiate human locomotion and modify it according to environmental and motivational demands (Nielsen, 2003). Perhaps the cortical activation that we observed in our stroke group, albeit reduced from normal, is adequate for initiating pedaling after which the maintenance of ongoing rhythmic movement occurs in the brainstem and/or spinal cord. The result may be the unsophisticated and minimally adaptable pattern of leg movement that is characteristic of stroke.

Finally, it is also possible that behavioral explanations account for our observations. During pedaling, the paretic and non-paretic limbs were mechanically coupled such that pedaling could have been accomplished by the non-paretic limb, with minimal contribution from the paretic limb. Thus, it is possible that the brain activation observed in the stroke group represented the volume required to produce the movement with one instead of both lower limbs. An asymmetric pedaling strategy, in which the non-paretic limb dominates task execution, may be simpler than a conventional strategy where both limbs contribute equally. Specifically, stroke survivors avoid the difficulty of activating the paretic limb, and they need not produce compensatory muscle activity with the non-paretic limb to correct for abnormal motor output of the paretic limb (Kautz and Brown, 1998; Schindler-Ivens et al., 2004, 2008; Fuchs et al., 2011). Also, if one leg is “driving” and the other is “riding,” task complexity is reduced by eliminating the need to coordinate the motor output of the two limbs. Thus, brain activation volume during pedaling in the stroke group may have been reduced due to reduced task complexity.

Several lines of evidence provide preliminary support for this behavioral explanation. First, stroke subjects displayed an asymmetrical pedaling strategy outside the scanner, wherein the non-paretic limb produced more than half of the mechanical work required to accelerate the crank (**Table 1**, %Work). Moreover, *post hoc* exploratory analyses showed that pedaling-related brain activation volume in stroke subjects tended to increase as the paretic limb contributed more of the work required to pedal (**Table 5**). The tapping data are also supportive of this behavioral explanation. During tapping, the paretic limb had to accomplish the work required for task completion without help from the non-paretic limb. In this case, brain activation volume was not reduced from normal and tended to be higher in stroke as compared to control subjects. Increased paretic limb activity, and possibly increased difficulty of using the paretic limb, may have elicited brain activation during tapping that was neither apparent nor required for pedaling, which did not require the paretic limb and was simplified through the predominant use of the non-paretic limb. Finally, preliminary work in our laboratory has shown that, in young controls, bilateral brain activation is apparent during unilateral pedaling and that unilateral pedaling produces more than half of the cortical activation volume associated with bilateral pedaling. Hence, the volume reductions observed here for stroke subjects are in line with observed volumes for unilateral pedaling. Hence, we have several indirect links between reduced brain activation volume and reduced contributions from the paretic limb. Future work should examine this issue by measuring and manipulating the work accomplished by the paretic limb while pedaling during fMRI. If our hypothesis is supported, brain activation would increase with increasing work of the paretic limb.

**Table 5:** Relationship between stroke-related impairment measures and volume of pedaling-related brain activation.

Type of measure	Name of measure	*r*	*p*
Clinical	Walking velocity	-0.194	0.546
	FM_LEtotal_	0.120	0.709
	FM_LEmotor_	0.060	0.852
	FM_LEsens_	0.166	0.605
Pedaling	%Work(+)	0.245	0.443
	%Work(-)	0.225	0.481
	%Work(net)	0.525	0.080
Walking	Temporal symmetry ratio (TSR)	-0.111	0.732
	Step length ratio (SLR)	0.151	0.639

We have also considered the possibility that reduced brain activation volume during pedaling post-stroke may represent improved efficiency in the neural strategy for controlling pedaling. Both groups pedaled successfully, and there was no between-group difference in pedaling rate. Thus, it would seem that fewer neural resources were used to produce the same movement, which is suggestive of improved efficiency. However, we suggest this possibility with the caveat that our criteria for matched performance were minimal. Namely, both groups were able to pedal, and there was no significant difference between groups in pedaling rate. We did not take into account other measures of performance, such as smoothness or the ability to respond to perturbations. The pedaling strategy in stroke subjects may not be smooth or adaptable. If this were the case, reduced neural activation would not reflect improved efficiency; rather, it may represent an association between inferior cortical drive and inferior performance. Future work should investigate this efficiency hypothesis by examining pedaling performance in more detail.

### Task-Dependency of Brain Activation Post-Stroke and Implications for Rehabilitation

Comparison of the tapping and pedaling data provide a preliminary indication that brain activation volume post-stroke is task-dependent and mutable, which may have important implications for rehabilitation. Our data show that portions of cortical and cerebellar tissue that were not active during pedaling became active during tapping. Hence, portions of the brain with the capacity for activation were inactive during pedaling. As indicated above, we suggest that the lack of activation in this tissue may reflect a different (and perhaps simpler) behavioral strategy for task completion. However, there may be serious limitations to this strategy. If both limbs do not make substantial contributions to functional movements of the lower limbs, movement will be clumsy and minimally adaptable. Fortunately for stroke survivors and rehabilitation professionals, our data also indicate that brain activation is task-dependent and mutable. Thus, it is realistic to expect that therapeutic interventions that increase use of the paretic limb may lead to enhanced cortical activation. Enhanced cortical activation may further enhance paretic limb use. Ultimately, this feedback loop could enhance paretic lower limb movement, resulting in better motor recovery. This framework is not unlike constraint induced movement therapy for the upper limb (Wolf et al., 2006). However, there are additional challenges associated with lower limb rehabilitation. Whereas many tasks involving the upper limb are performed unilaterally, walking and other functional lower limb movements require simultaneous and coordinated movement of both legs. Thus, when considering rehabilitation techniques that require paretic lower limb use, one must also consider the effects on bilateral coordination. Our pedaling paradigm can help examine this issue, as the supine nature of the task allows us to reduce or eliminate use of the non-paretic limb without concerns about balance or body weight support. Moreover, the fMRI-compatibility of our paradigm permits examination of brain activation associated with differing levels of paretic limb activity. Tests of these hypotheses are forthcoming from our lab with fMRI studies comparing brain activation post-stroke during unilateral pedaling and bilateral pedaling without mechanical coupling of the two legs. Results may have important implications for rehabilitation of hemiparetic walking, as reduced use of the paretic leg also occurs during walking (Allen et al., 2011; Raja et al., 2012).

### Additional Methodological Implications

Before concluding, we will discuss some additional methodological issues that the limit the interpretation of our data and suggest the need for additional study.

While beneficial in many ways, analyzing our data in native space limited the spatial resolution or our analysis. Recall that data were analyzed in native space because anatomical landmarks for standardization were not always available in stroke subjects. Hence, group analysis in standard space would have required estimation of the landmarks, leading to errors in localization of brain activation. The use of native space allowed us to examine brain activation in a heterogeneous sample of stroke survivors, some with large lesions affecting considerable brain tissue. Moreover, the native space approach permitted direct, between-group comparisons of the same brain regions because all variables were extracted from the same anatomical regions in all subjects, namely M1/S1, BA6, and cerebellum (vermis, lobules IV, V, VIII). However, with respect to spatial resolution, this approach is limited. Our analysis allowed us to determine that, across groups, the same brain regions were active and that the volume of activation within regions was smaller in the stroke group, as compared to control. Reduced volume post-stroke implies that both groups did not activate the same portion of each region. However, we do not know what portion failed to activate in the stroke group. For example, there could have been a decrease in the spatial extent of activation around the same center of activation. Alternatively, a particular portion of a region may have failed to activate in the stroke group, causing a shift in the center of activation. The use of native space also limits our understanding of brain activation in BA6. When we delineated the regions of interest, we did not divide BA6 into its component parts (i.e., supplemental motor area and premotor cortex). Our earlier work in young, healthy controls showed pedaling-related brain activation in supplemental motor area, but not premotor cortex (Mehta et al., 2012). Here, we do not know whether activation in the stroke group was also limited to supplemental motor area. In light of the limitations of native space, it would be useful for future work to constrain the stroke group to subjects with lesions that do not affect landmarks for standardization. This approach would allow us to standardize the brains, flip the images to get all the lesions on the same side, create group activity maps, and perform group analysis with *t*-tests, contrasts, and x, y, z coordinates. This approach would permit a better spatial description of activation and might reveal shifts in the location of activation within regions.

The exclusion of stroke subjects with M1 and S1 lesions is another methodological factor that may have had an important influence on our results. Individuals with M1 or S1 lesions might have shown different brain activation patterns than those observed here. For example, it is possible that M1 or S1 lesions could result in over activation of the non-lesioned cortex and/or activation of brain regions not typically involved in the movement. Indeed, in planning this work, we anticipated such findings, which was a major reason for *excluding* patients with M1 and S1 lesions. It is also the reason that we included the tapping task. We were concerned that the fMRI signal associated with pedaling would be so unusual in the stroke group that we would not know if the signal represented authentic task-related brain activation or if it was artifact. Similarly, extensive remapping of the leg areas of the cortex might have been interpreted as artifact. In selecting subjects without M1 or S1 lesions, we reasoned that tapping (and probably pedaling) would elicit activation of these regions. The presence of somatotopically predictable brain activation would provide confidence in our technique. On this count, we succeeded. We saw M1 and S1 activation near the midline of the cortex, in similar locations for pedaling and tapping. Thus, our fMRI signals appear to represent task-related brain activation. However, with respect to detecting compensatory changes in brain activation and/or between-group differences in brain function, we are limited. Alterations in brain activation may not be required when M1 and S1 remain intact. Importantly, we are not suggesting that over activation, atypical activation, and/or remapping do not exist after stroke. Compensatory activations during pedaling may be related to the location and extent of brain injury. Loss of tissue in the leg area of M1 and S1 may be an important trigger for such changes. Now that we have established the validity of our technique, future work should examine the influence of lesion location, including lesions in M1 and S1, on pedaling-related brain activation.

Also with respect to compensatory brain activation, our technique for identifying active regions is another important consideration. Specifically, we ignored inconsistent activations that were distinctive of individual subjects, and we analyzed only brain regions that were consistently active across subjects. This approach may have caused us to overlook important activations outside M1/S1, BA6, and cerebellum and may be one reason that we did not observe atypical activations. However, this approach also ensured that we did not over interpret inconsistent activations that were unique to individuals. Moreover, we were still able to examine between-group differences in activation in brain regions typically involved by pedaling, as our prior work in young, healthy controls showed that pedaling-related brain activation was limited to M1/S1, BA6, and cerebellum (Mehta et al., 2009, 2012). Nevertheless, future work should address this issue by limiting the sample of stroke subjects to individuals with intact landmarks so that standardization and group averaging is possible. Whole-brain, group analysis might reveal compensatory brain activation during pedaling post-stroke.

## Conclusion

We demonstrated that pedaling can be used with fMRI to examine brain activation associated with lower limb movement in people with stroke and age-matched controls. Preliminary observations also suggest that brain activation volume during pedaling is reduced in people post-stroke, as compared to controls. Reduced brain activation volume was task-dependent and mutable; it may be due to anatomical, physiological, and/or behavioral changes post-stroke, but methodological influences cannot be excluded. Future work will use our novel fMRI pedaling paradigm to further examine brain activation post-stroke during continuous, reciprocal, lower limb movement in order to understand how the brain adapts – or fails to adapt – post-stroke to produce these movements.

## Author Contributions

All the authors contributed to the design and interpretation of the work; all approved the final version. SS-I and NP collected and analyzed data and wrote the manuscript.

## Conflict of Interest Statement

The authors declare that the research was conducted in the absence of any commercial or financial relationships that could be construed as a potential conflict of interest.
